# Atrial Fibrillation and Early Vascular Aging: Clinical Implications, Methodology Issues and Open Questions—A Review from the VascAgeNet COST Action

**DOI:** 10.3390/jcm13051207

**Published:** 2024-02-20

**Authors:** Giacomo Pucci, Andrea Grillo, Kalliopi V. Dalakleidi, Emil Fraenkel, Eugenia Gkaliagkousi, Spyretta Golemati, Andrea Guala, Bernhard Hametner, Antonios Lazaridis, Christopher C. Mayer, Ioana Mozos, Telmo Pereira, Dave Veerasingam, Dimitrios Terentes-Printzios, Davide Agnoletti

**Affiliations:** 1 Unit of Internal Medicine, Santa Maria University Hospital, 05100 Terni, Italy; 2 Department of Medicine and Surgery, University of Perugia, 06125 Perugia, Italy; 3 Department of Medicine, Surgery and Health Sciences, University of Trieste, 34149 Trieste, Italy; 4 Biomedical Simulations and Imaging (BIOSIM) Laboratory, School of Electrical and Computer Engineering, National Technical University of Athens, 15780 Athens, Greece; 5 1st Department of Internal Medicine, Faculty of General Medicine, Pavol Jozef Šafárik University, 04011 Košice, Slovakia; 6 3rd Department of Internal Medicine, Aristotle University of Thessaloniki, Papageorgiou General Hospital, 54124 Thessaloniki, Greece; 7 Medical School, National and Kapodistrian University of Athens, 10675 Athens, Greece; sgolemati@med.uoa.gr; 8 Vall d’Hebrón Research Institute (VHIR), 08035 Barcelona, Spain; 9 CIBER CV, Instituto de Salud Carlos III, 28029 Madrid, Spain; 10 AIT Austrian Institute of Technology, Center for Health & Bioresources, Medical Signal Analysis, 1210 Vienna, Austria; 11 Department of Functional Sciences—Pathophysiology, Center for Translational Research and Systems Medicine, “Victor Babes” University of Medicine and Pharmacy, 300173 Timisoara, Romania; 12 H&TRC—Health & Technology Research Center, Coimbra Health School, Polytechnic University of Coimbra, 3000-331 Coimbra, Portugal; 13 Laboratory for Applied Research in Health (Labinsaúde), Polytechnic University of Coimbra, 3000-331 Coimbra, Portugal; 14 Department of Cardiothoracic Surgery, Galway University Hospitals, H91 YR71 Galway, Ireland; 15 First Department of Cardiology, Hippokration Hospital, Medical School, National and Kapodistrian University of Athens, 11527 Athens, Greece; 16 Cardiovascular Internal Medicine, IRCCS Azienda Ospedaliero—Universitaria di Bologna, 40138 Bologna, Italy; davide_agnoletti@hotmail.com; 17 Cardiovascular Internal Medicine, Medical and Surgical Sciences Department, University of Bologna, 40138 Bologna, Italy

**Keywords:** vascular aging, atrial fibrillation, arteriosclerosis, cardiovascular disease, endothelial dysfunction, arterial stiffness, pulse wave velocity, flow mediated dilation

## Abstract

Atrial fibrillation (AF), the most common cardiac arrhythmia, is associated with adverse CV outcomes. Vascular aging (VA), which is defined as the progressive deterioration of arterial function and structure over a lifetime, is an independent predictor of both AF development and CV events. A timing identification and treatment of early VA has therefore the potential to reduce the risk of AF incidence and related CV events. A network of scientists and clinicians from the COST Action VascAgeNet identified five clinically and methodologically relevant questions regarding the relationship between AF and VA and conducted a narrative review of the literature to find potential answers. These are: (1) Are VA biomarkers associated with AF? (2) Does early VA predict AF occurrence better than chronological aging? (3) Is early VA a risk enhancer for the occurrence of CV events in AF patients? (4) Are devices measuring VA suitable to perform subclinical AF detection? (5) Does atrial-fibrillation-related rhythm irregularity have a negative impact on the measurement of vascular age? Results showed that VA is a powerful and independent predictor of AF incidence, however, its role as risk modifier for the occurrence of CV events in patients with AF is debatable. Limited and inconclusive data exist regarding the reliability of VA measurement in the presence of rhythm irregularities associated with AF. To date, no device is equipped with tools capable of detecting AF during VA measurements. This represents a missed opportunity to effectively perform CV prevention in people at high risk. Further advances are needed to fill knowledge gaps in this field.

## 1. Introduction

Atrial fibrillation (AF), the most common sustained cardiac arrhythmia, is associated with a high burden of cardiovascular (CV) morbidity and mortality, mainly related to an increased risk of cardioembolic stroke and heart failure [1]. The global cumulative mortality attributed to AF was 0.51% in 2017, reflecting an 81% relative increase over the past two decades [1]. The prevalence of AF is currently increasing and is expected to rise in the coming years across all age groups and regions [2]. This is primarily attributed to the growing burden of comorbidities, socioeconomic deprivation and AF risk factors such as hypertension, obesity, diabetes and ischemic heart disease [3]. 

From a pathophysiological point of view, AF is defined as a supraventricular tachyarrhythmia marked by uncoordinated atrial electrical activation, leading to ineffective atrial contraction and causing an irregular heart rhythm. From a clinical perspective, AF is classified as paroxysmal (PAF, episodes lasting less than one week), persistent (continuously sustained beyond 7 days, including episodes terminated by cardioversion) or permanent (stable AF rhythm with no further attempts to restore/maintain sinus rhythm) [4]; long-standing persistent AF (continuously sustained for an extended period, typically lasting beyond 12 months); valvular/non-valvular AF (valvular AF indicates the presence of moderate/severe mitral stenosis or a mechanical prosthetic heart valve(s)). The classification of lone AF, referring to AF without any other cardiorespiratory diseases or risk factors, is now dismissed.

Notably, asymptomatic AF poses a challenge to clinicians, potentially causing delays in establishing preventive strategies [5]. It is estimated that one out of ten ischemic strokes is related to a previously unknown history of AF [6]. This could be prevented by implementing digital systems and mobile health technologies for AF screening and detection, especially in individuals at risk [7].

The term vascular aging (VA) is commonly used to describe the deterioration of both structural and functional components of the arterial tree, although a universally acknowledged definition is still lacking [8]. 

### 1.1. Structural Arterial Properties: The Arterial Stiffness

At a structural level, the process of VA is identified with the progressive stiffening of the arterial tree, namely arterial stiffness (AS). This process mainly occurs at the level of large elastic arteries such as the aorta and the carotid arteries, where a mechanical remodeling of the arterial wall is observed [9]. The most commonly used method for the non-invasive estimation of arterial stiffness is the measure of the pulse wave velocity (PWV), which represents the velocity of the pressure waves generated from the systolic contraction along a defined arterial segment. Most commonly, the carotid–femoral PWV (cfPWV) is used as a marker of aortic stiffness. CfPWV has been associated with adverse clinical outcomes in several population settings [10], and predicts CV outcome better than chronological aging [11,12]. Several other methods used for arterial stiffness estimation are summarized in Table 1.

### 1.2. Functional Arterial Properties: The Endothelial Dysfunction

At a functional level, the hallmark of VA is the impairment of endothelial function, which is the result of a decrease in nitric oxide synthase (eNOS) expression in endothelial cells and that, in turn, promotes the development of a prothrombotic state [13] and atherosclerosis [9]. This process, namely the endothelial dysfunction (ED), is hastened by oxidative stress and occurs in response to both physiological aging and systemic inflammation [14,15]. Flow-mediated dilation (FMD), usually assessed at the brachial artery, has been established as a reliable and reproducible technique for assessment of ED [16,17], and has been independently associated with vascular disease and adverse CV events [18]. 

The exposure to CV risk factors, including smoking, obesity, hypertension, diabetes and hypercholesterolemia, promotes the development of both early VA and AF. Therefore, measurement of VA biomarkers such as PWV and brachial FMD in people with AF, or at risk for it, has a strong rationale and large expected impact on clinical practice to better characterize the individual CV risk and to provide targeted interventions.

However, there are some issues undermining the measurement of VA biomarkers, especially in patients with AF. First, the changing of heart period and stroke volume brings questions regarding the accuracy of measurement of VA biomarkers. The measure of PWV by sequential tonometry in a given arterial segment is exposed to an increased variability in the time it takes for the pressure wave to travel between two points. Additionally, changes in stroke volume can also affect measurements based on pulse volume and flow detection, such as FMD. For this reason, measurement of VA in patients with AF is underused and little is known about the prognostic value of VA biomarkers in patients with AF. 

With all these premises, a network of scientists and clinicians from the COST Action VascAgeNet (CA18216) [19] identified a list of five clinical and practical key questions regarding the relationship between VA biomarkers and AF and critically reviewed the literature, with a special focus on studies using PWV and ED measures as reference methods for the evaluation of structural and functional arterial properties, in order to find potential answers. The list of question is the following:

1. Are VA biomarkers associated with AF?
2. Does early VA predict AF occurrence better than chronological aging?
3. Is early VA a risk enhancer for the occurrence of CV events in AF patients?
4. Are devices measuring VA suitable to perform subclinical AF detection?
5. Is the measurement of VA negatively influenced by AF-related rhythm irregularities?

## 2. Materials and Methods

**Figure 1:**
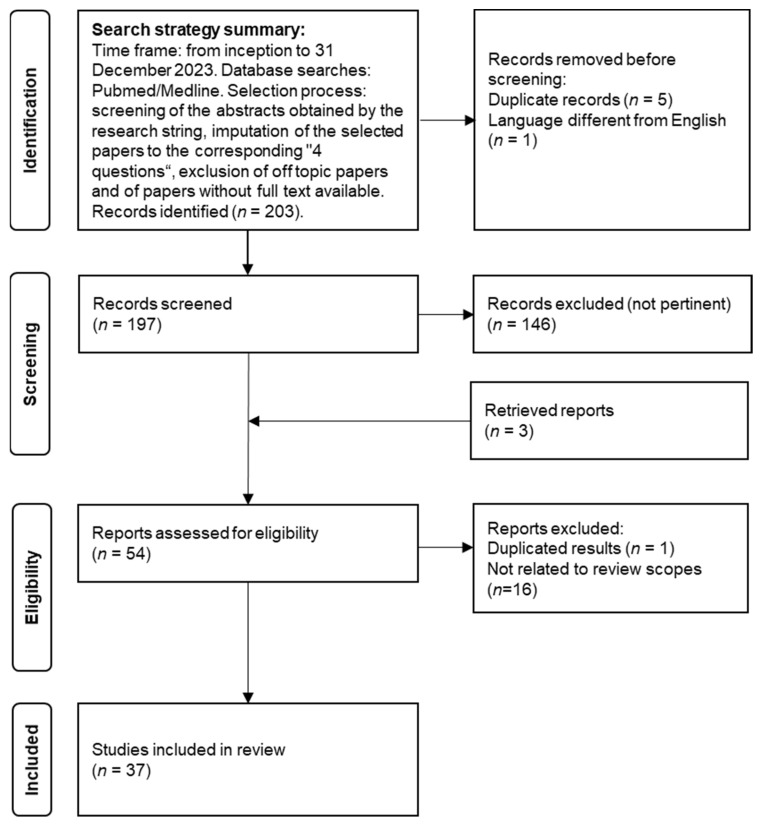
Flow chart of the review process.

The search was performed using PubMed/MEDLINE databases with relevant keywords on the topics. We selected peer-reviewed articles published from inception to 31 December 2023. Papers written in languages other than English, not pertinent to the present review or whose full text was not available were excluded. The complete search string incorporated inclusive keywords on VA (e.g., “vascular ageing”, “vascular aging”, “vascular senescence”), arterial stiffness (e.g., “arterial stiffness”, “arterial compliance”, “pulse wave velocity”, “PWV”, “augmentation index”, “AIx”, “central blood pressure”, “pulse pressure”), subclinical atherosclerosis (“carotid intima-media thickness”), ED (“flow mediated dilation”) and inclusive keywords on AF (“atrial fibrillation”, “paroxysmal atrial fibrillation”, “persistent atrial fibrillation”). The pertinent papers were evaluated and eventually included in the final manuscript. We considered all papers in open-access and non-open-access journals. A flow chart of the review process, the search strategy summary and the checklist for the narrative review are provided in Figure 1 and Table S1 (Supplementary Materials).

## 3. Results

From the 203 papers identified, we included 37 papers, offering an overview of the current literature. Practical recommendations for the use of VA measures in the context of AF were formulated in agreement between the authors and are presented in italics at the beginning of each paragraph.

**Figure 2:**
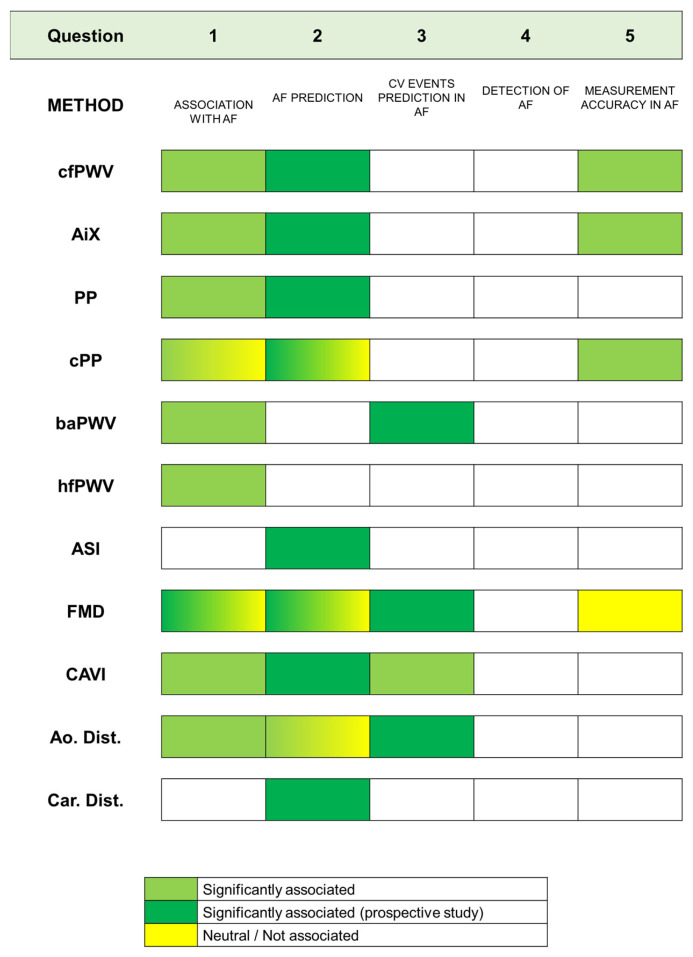
Results of included studies according to methods and the five questions considered in the paper.

The included studies are organized in Table 2, and their results according to method are summarized in Figure 2. For more clarity, a detailed description of each VA biomarker included in the present study is provided in Table 2.

### 3.1. Question 1—Are VA Biomarkers Associated with AF?

*Answer: There is substantial evidence, although partly derived from small studies, that subjects with AF have early VA compared to subjects in sinus rhythm. This association is largely explained by concomitant CV risk factors, such as hypertension, dyslipidemia and diabetes mellitus, which are often present in people with AF. However, at least for measures of AS, there is also evidence that this association remains significant after multiple adjustment.*

In a case–control study, 76 patients with either permanent or paroxysmal AF were compared to a control group of 75 healthy individuals. Compared to patients in sinus rhythm, patients with AF had higher PWV (8.0 m/s vs. 7.2 m/s, *p* < 0.001), central SBP (118 mm Hg vs. 114 mm Hg, *p* = 0.033), central PP (39 mm Hg vs. 37 mm Hg, *p* = 0.035) and lower PP amplification (PPA), measured as the ratio between peripheral and central PPs (1.24 vs. 1.30, *p* = 0.015). The relationship between cfPWV and AF remained significant after adjustment for age, sex, heart rate, weight, MAP and glomerular filtration rate [44]. In a large cross-sectional study conducted on Japanese men and women (*n* = 4264, age range 40–79 years), the PPA was negatively associated with the prevalence of AF and total arrhythmia, independently of CV risk factors. In a multivariate model adjusted for age, sex, BMI, heart rate, SBP, smoking, alcohol consumption, serum total and HDL cholesterol, triglycerides, diabetes mellitus and use of antihypertensive and lipid-lowering medications, as compared with subjects in the highest tertile of PPA, subjects in the lower tertile of PPA showed higher odds of having AF (OR 3.4, 95%CI 1.4–8.6). No significant associations between either brachial or central PP and AF prevalence were reported [45]. 

In a study by Doi et al., 122 patients with PAF were compared with 122 age- and sex-matched controls without PAF. All subjects were in sinus rhythm. AIx was calculated from the radial artery waveform using applanation tonometry. After adjustment for age, sex, heart rate and medications, AIx was significantly higher in patients with than without PAF (89 ± 1.0 vs. 82 ± 1.0%, *p* < 0.001). Each 10% increase in AIx was associated with higher odds of PAF (OR 1.6, 95%CI 1.13–2.25) [46]. These data suggest that AF remains associated with increased arterial stiffness even after restoration of sinus rhythm.

Similarly, other measures of AS, such as baPWV and heart–femoral PWV (hfPWV), were found to be higher in subjects with AF than in controls, independently of confounders. The former (baPWV) was evaluated in a population of 132 patients with hypertension and AF (78 with PAF and 84 with persistent AF) compared to 136 patients with hypertension in sinus rhythm. In a multivariate logistic regression model adjusted for multiple CV risk factors, each unit increase in baPWV corresponded to 10.4% increased risk of having AF. Interestingly, this association was no longer significant after further adjustment for uric acid, suggesting that this factor could be implicated in the mechanism of AF development in the presence of early VA [47]. The latter (hfPWV) was found to be higher in 35 subjects with AF compared to 33 subjects in sinus rhythm (1028 ± 222 vs. 923 ± 110 cm/s, *p* = 0.03). Together with age and systolic BP, the presence of AF was an independent predictor of increased hfPWV [48].

AS, measured by CAVI, also showed an association with AF. When 91 patients with PAF, after restoring sinus rhythm, were compared with 90 age- and sex-matched subjects without PAF, CAVI was significantly higher in the former compared to the latter group (9.0 ± 1.0 vs. 8.7 ± 0.8, *p* < 0.01). This difference, even if clinically small, remained significant after adjustment for age, gender, heart rate and use of antihypertensive and antiarrhythmic drugs [49]. In a study conducted in 33 subjects (mean age 73 ± 12 years) with persistent AF undergoing external cardioversion, CAVI was inversely correlated with AF duration, independently of age and cardiac chamber dimensions [50]. CAVI also showed a correlation with AF in large cross-sectional studies, such as the study by Chung et al. that enrolled 8048 subjects screened for CV disease who underwent electrocardiogram and CAVI. The prevalence of AF was significantly higher in the high group (2.2% in subjects with CAVI ≥ 8) compared with the low group (1% in subjects with CAVI < 8, *p* < 0.001). Multivariate analysis further depicted the association of CAVI ≥ 8 with AF prevalence as independent of age, sex and CV risk factors (OR = 2.06, 95%CI 1.40–3.05, *p* < 0.001). The association of CAVI with AF was also evaluated in subgroups stratified according to the Framingham risk score. Higher odds were found in people at intermediate (OR 3.06, 95%CI 1.39–6.74) and high (OR 3.88, 95%CI 1.14–13.17) CV risk [51]. In 164 subjects with AF, compared to 652 controls after propensity score matching, significantly higher odds of AF were found at each 1-unit increase in CAVI (OR 1.37, 95%CI 1.08–1.22, *p* = 0.008) [30]. 

Several studies focused on the association between ED and AF. Subjects younger than 60 years with AF and without any other CV risk factor (defined as lone AF, *n* = 43), compared to age- and sex-matched controls (*n* = 51), showed significantly lower values of FMD (5.8 ± 3.9% vs. 7.6 ± 4.4%, *p* = 0.04) [52]. In subjects with AF undergoing restoration of regular sinus rhythm, brachial FMD did significantly improve after cardioversion (0.32 ± 0.07 mm during AF, 0.42 ± 0.08 mm after cardioversion, *p* < 0.01). In 10 patients who underwent a further AF relapse, FMD returned to pre-cardioversion values (0.33 ± 0.07 mm, *p* < 0.05 vs. post-cardioversion) [53]. Another study observed a short-term improvement in FMD, which was observed in 32 patients after 24 h of restoration of sinus rhythm (FMD during AF rhythm 8.4 ± 3.8%, FMD after 24 h of sinus rhythm restoration 10.7 ± 3.9%, *p* < 0.001) [21].

Although these findings support the hypothesis of an independent association between ED and AF, they are, however, counterbalanced by results from other studies. Indeed, in a large cohort study conducted in a sample of 15,010 individuals from the general population, the odds of having reduced FMD in patients with AF (*n* = 466, 3.1% of the population) was no longer significant after multiple adjustment to age, sex, heart rate, BMI, diabetes, smoking status, LDL/HDL cholesterol ratio and SBP (OR 1.03, 95%CI 0.88–1.21, *p* = 0.59) [54]. 

Long duration of arrythmia and the frequency of AF episodes showed, in some studies, some degree of association with worsening endothelial function. In a study by Khan et al. [55], ED measured by brachial FMD was significantly different between 30 subjects with permanent AF vs. 31 subjects with PAF (3.1% vs. 5.9%, *p* = 0.02). Contrary to FMD, nitroglycerine-mediated vasodilation (NMD), a measure of endothelium-independent vasoreactivity, seems not to be affected by AF. In 38 subjects with lone AF compared to 28 healthy controls matched by age, gender and atherosclerotic risk factors, no difference between groups were found in terms of NMD [56]. Similar results were observed in another study where endothelium-independent vasodilation did not change after sinus rhythm restoration by cardioversion in 46 patients with AF [21].

### 3.2. Question 2—Does VA Predict the Occurrence of AF Better Than Chronological Aging?

*Answer: The role of arterial stiffness as an independent predictor of AF incidence is supported by the results of large-scale prospective observational studies and Mendelian randomization studies. A variety of markers of VA, including cfPWV, augmentation pressure and AIx, CAVI, elevated central and peripheral PP and aortic and carotid distensibility, showed (chronological) age-independent associations with the future occurrence of AF. In many cases, however, this association was mediated by increased BP levels which could, at least in part, confound the association between VA and AF. Arterial stiffness was also an independent predictor of AF recurrences after restoration of sinus rhythm. Associations were also found between arterial stiffness and features of cardiac remodeling, such as left atrial enlargement, pathophysiologically linked to a higher risk of AF incidence. The evidence in favor of FMD as a risk factor for AF incidence is weaker and partly counterbalanced by negative findings.*

Results from three large-scale, population-based, cohort studies (the Atherosclerosis Risk in Communities, ARIC, Study, *n* = 13,907, the Multi-Ethnic Study of Atherosclerosis, MESA, *n* = 6640, and the Rotterdam Study, *n* = 5220) investigated the prognostic ability of measures of VA in predicting the future occurrence of AF [27]. All these studies adopted carotid distensibility as a marker of AS. In the Rotterdam Study and in a subcohort of the ARIC study, measures of cfPWV were also available. Concerning carotid distensibility, in multivariate models adjusted for multiple confounders including age, ethnicity, use of antihypertensive medication, current smoking, diabetes, history of heart failure and history of myocardial infarction, the hazard ratios (HR) associated with a 1 SD increase in carotid distensibility were 0.90 (95%CI 0.83–0.97, *p* < 0.001) in the ARIC Study and 0.83 (95%CI 0.70–0.98, *p* < 0.001) in the MESA. However, the results became not significant when height, weight, systolic and diastolic BP were included in the models (both *p* > 0.05). In the Rotterdam Study, the HR of AF associated with carotid distensibility was no longer significant after multiple adjustment (0.98, 95%CI 0.83–1.15, *p* = 0.78). In both cases, the loss of significance in multivariate models after adjustment for BP could be attributed to the functional influence of BP values on PWV. Indeed, PWV is consistently dependent on the wall stretch caused by the distending pressure and the passive loss of compliance of the arterial wall [57].

Similarly, cfPWV in the Rotterdam Study showed an independent association with AF incidence (HR 1.15, 95%CI 1.03–1.29, *p* = 0.016 per 1 SD increase in cfPWV), which was lost when the model was adjusted for BP [56]. In the ARIC Study, cfPWV demonstrated a U-shaped association with AF risk: in Cox regression models adjusted for age, race, center, sex, education levels and hemodynamic and clinical factors, the HR for incident AF in the first, third and fourth quartiles were 1.49 (95%CI 1.06–2.10), 1.59 (95%CI 1.14–2.10) and 1.56 (95%CI 1.10–2.19), respectively, compared to those in the second quartile, which was taken as a reference [28].

The predictive role of arterial stiffness for AF incidence was also analyzed in the Framingham Heart Study offspring and third-generation cohorts. Among 5797 participants (mean age 61 ± 10 years) followed up for an average period of 7.1 years, cfPWV, AIx and cPP were all univariately associated with increased risk of AF incidence [29]. In fully adjusted models, only AIx remained significantly associated with AF incidence (HR 1.16, 95%CI 1.02–1.32). 

In a cohort study conducted in a Japanese population (*n* = 5418), baseline CAVI values ≥ 8.0, along with age ≥ 65 years and male sex, were found to independently predict the incidence of AF (*n* = 22, 0.41%) over 4 years (HR 5.27, 95%CI 1.6–17.3) [30]. 

In a subcohort of the MESA (3441 participants aged 45–84 years followed up for 7.8 years), high pulse pressure and low aortic distensibility measured by magnetic resonance imaging (MRI) were both univariately associated with the development of AF. In a multivariate analysis, after adjustment for age, sex, ethnicity, education, height, body mass index, smoking status, antihypertensive treatment, diabetes, left ventricular mass, heart rate and MAP, and after excluding aortic distensibility outliers, only PP remained significantly associated with AF risk, whereas aortic distensibility lost its significance. Each 1 SD increase in PP was independently associated with a 45% increased risk of AF (HR 1.45 95%CI: 1.13–1.87, *p* = 0.004) [31]. Increased PP independently predicted incident AF also in 350 patients with type 2 diabetes who were free from AF at baseline who were followed up for 10 years (adjusted OR: 1.76 for each SD increment, 95%CI 1.1–2.8, *p* = 0.01) [29]. The Losartan Intervention for Endpoint Reduction in Hypertension Study (LIFE Study) included 9193 patients with essential hypertension and electrocardiographic LV hypertrophy followed up for a period of 5 years. Increased brachial PP, either baseline or in treatment, was independently associated with a higher risk of new-onset AF in multivariate Cox regression analysis (HR per 10 mmHg baseline PP increase: 1.24, 95%CI 1.14–1.35, HR per 10 mmHg in-treatment PP increase 1.21, 95%CI 1.11–1.33 for in-treatment PP, both *p* < 0.001). PP was equivalent to SBP and DBP in predicting new-onset AF, but when included in the same statistical model, PP was demonstrated to be the strongest predictor [33]. In another study from the Framingham Heart Study cohort, the predictive power of increased PP for AF incidence was evaluated in a large general population including 5331 individuals followed for 12 years. After adjustment for a substantial number of confounders, the hazard ratio of new-onset AF associated with a 20 mmHg PP increase was 1.26 (95%CI 1.12–1.43, *p* < 0.001) [32]. It is worth noting that: (i) the increase in PP is dependent on both the physiological aging process and the associated increase in arterial stiffness induced by CV risk factors; (ii) an increase of 20 mmHg in PP can be observed over the lifespan only after several decades [58].

The prognostic superiority of central over peripheral BP measurement in incident AF was also observed in a predominantly older population-based cohort including 769 participants in sinus rhythm with no history of AF or stroke (mean age 70.5 years). Over 9.5 years, AF occurred in 83 participants. No peripheral BP value showed a significant association with incident AF. By contrast, after adjustment for age, sex, race and the number of antihypertensive drugs, both central SBP (HR 1.12 for 10 mmHg increment, 95%CI 1.00–1.25, *p* = 0.047) and central PP (HR 1.16 for 10 mmHg increment, 95%CI 1.00–1.34, *p* = 0.048) showed predictive value for AF incidence [34]. These results are of importance given that central PP, rather than peripheral PP, is more strongly linked to the age-related stiffening of large arteries [59].

In a cohort of 151 patients (mean age 71.9 years, mean follow-up 21 months) with AF, who restored sinus rhythm after pulmonary vein isolation, AS, evaluated by aortic distensibility (AD) of the descending aorta using transesophageal echocardiography, was found to be an independent predictor of AF recurrence (OR 3.6, 95%CI 2.8–4.1) [36]. In a study including 68 patients with AF who underwent a successful catheter ablation procedure, higher AF recurrence rates during a mean follow-up of 3 years were found in patients with higher values of peripheral PP, central PP and augmented pressure [37]. Among 31 older patients (mean age 78 ± 7 years) undergoing electrical cardioversion, CAVI was directly related to the risk of AF recurrence: for each one-unit increase in CAVI, the HR for AF recurrence was 2.31 (95%CI 1.01–5.25) [38]. However, in these two latter cases, the regression models were not adjusted for relevant confounders (e.g., age). 

In a cohort of 103 patients with PAF, compared to age- and sex-matched controls, left atrial diameter was significantly correlated with augmented pressure and AIx (both *p* < 0.001). Interestingly, left atrial diameter was the only independent predictor of AF recurrences following cardiac ablation over a follow-up period of 6 months [39]. 

A genome-wide association study, with Mendelian randomization including 225,636 participants from the UK Biobank, demonstrated a significant association between genetically determined increased levels of a photoplethysmography-derived arterial stiffness index (ASI) and the incidence of AF (OR, 1.8 per SD ASI phenotype, 95%CI, 1.4–2.2) [40]. This Mendelian randomization approach provides evidence of the causal inference between arterial stiffness and AF. 

#### Indirect Markers of AF

AS was frequently found to be associated to well-established cardiac markers associated with increased risk of developing AF, such as LV hypertrophy, LV diastolic dysfunction and elevated LV filling pressure that, in turn, can result in an elevated left atrial pressure leading to left atrial dilation. In a study including 43 younger patients (aged 46 ± 8 years), with moderate to severe obstructive sleep apnea, a cfPWV > 10 m/s significantly correlated with left atrial diameter (r = 0.45; *p* < 0.001) both in univariate and multivariate analysis [41]. In a study conducted on 310 middle-aged hypertensive patients, cfPWV and elevated PP measured over 24 h were significantly and directly associated with left atrial diameter (r = 0.27 and r = 0.32, respectively, both *p* < 0.001) even after adjustment for age, sex, body mass index, indexes of LV structure and geometry and filling pressure [42]. However, regarding the limitations of echocardiographic assessment, which is prone to measurement errors, we should consider that the LA overload is reasonably mediated by LV alterations. 

The role of ED in predicting AF incidence was evaluated in a subcohort of 3921 (mean age 58 ± 9 years) participants of the Framingham Heart Study. In this study, FMD was negatively associated with the future occurrence of AF in univariate and multivariate analyses (adjusted HR 0.79, 95%CI 0.63–0.99) [27]. Contrasting results were observed in a cohort of 2027 old individuals enrolled in the Cardiovascular Health Study (mean age 78.3 years). Over a median follow-up of 11 years, 754 incident AF cases occurred. After adjustment for age, sex, race, height, weight, CV disease, cigarette smoking, hypertension, diabetes, kidney function, C-reactive protein, physical activity, alcohol consumption and statins, the risk of AF did not differ according to baseline FMD (HR per FMD unit increment 1.01, 95%CI 0.97–1.05) [43]. Therefore, in comparison to positive findings observed in the Framingham Heart Study, results from this study suggested that, at least in older individuals, the utility of brachial FMD as a risk marker for AF was minimal.

### 3.3. Question 3—Is Early VA a Risk Enhancer for the Occurrence of CV Events in AF Patients? 

*Answer: The prognostic impact of measures of VA on future CV events in patients with AF has been tested only in small-scale, short-term, longitudinal studies and remains a matter of investigation. Data are also limited concerning the role of ED as a risk enhancer for adverse CV events in AF patients. Data from studies using changes in surrogate markers of CV prognosis as the primary endpoint are also extremely scarce.*

Chen et al. assessed arterial stiffness by measuring brachial–ankle PWV (baPWV) in 167 patients with persistent AF. After a median follow-up of 26 months, the authors found that high baPWV was independently associated with an increased risk of a composite outcome including CV death, non-fatal stroke and myocardial infarction and hospitalization for heart failure. This association remained significant after adjusting for multiple CV risk factors (HR = 1.150; 95%CI: 1.034–1.279, *p* = 0.01). Most importantly, they demonstrated that baPWV had an incremental value in CV outcome prediction, pointing towards the usefulness of this marker in the risk stratification of these patients [22]. In another study, arterial stiffness was assessed by aortic distensibility during transesophageal echocardiography in 151 patients with AF before successful restoration of sinus rhythm with pulmonary vein isolation. Fifty-four controls with similar CV risk profile were also enrolled in the study. Results showed that, after a median follow-up of 21 months, decreased aortic distensibility was univariately associated with a composite endpoint that included AF recurrences, stroke, acute decompensated heart failure, cardiovascular and all-cause hospitalizations. Subjects in the lowest quartile of aortic distensibility showed an increased number of composite events as compared to those in the third quartile (*p* = 0.03) and in the fourth quartile (*p* = 0.001) [23].

The association between arterial stiffness and future CV event was also assessed using indirect descriptors of future CV events in AF. In a cohort of 117 patients with paroxysmal or persistent AF compared to 274 controls, cfPWV was independently associated with plasma N-terminal pro-B-type natriuretic peptide (NT-proBNP), which is considered a surrogate marker of CV prognosis (β = 0.234; 95%CI: 0.100–0.367, *p* = 0.001). Interestingly, only in patients with paroxysmal or persistent AF were increased values of cfPWV related to greater NT-proBNP plasma levels, whereas this was not observed in the control group, suggesting a relationship between AF, increased arterial stiffness and adverse CV outcomes [24]. 

The prognostic significance of ED, measured by FMD, in AF patients was evaluated in a cohort of 514 individuals with AF followed up for an average period of 24 months. A composite endpoint of CV events, defined as the occurrence of stroke/transient ischemic attack, myocardial infarction, urgent revascularization and CV death, occurred in 44 patients. In a Cox proportional hazards analysis, after multiple adjustment for other CV risk factors such as MI, history of stroke/TIA, heart failure, treatment with statins, smoking habits, gender and age, individuals with an FMD below 4.6% were at increased risk of CV events (HR 2.20 95%CI 1.13–4.28, *p* = 0.020) [25]. In another prospective observational study, FMD was measured by ultrasound in 291 patients with a positive history of PAF lasting no longer than six months. After a mean follow up of 33 months, subjects with FMD lower than 5.9% showed a doubled rate of composite adverse CV events, which included cardiovascular death, non-fatal myocardial infarction, stroke and heart failure hospitalization (37.1% versus 18% in patients with FMD > 5.9%, *p* < 0.001), which remained significant after adjustment for classical CV risk factors (HR: 3.036, 95%CI 1.546–5.963, *p* = 0.01) [26].

### 3.4. Question 4—Are Devices Measuring VA Suitable to Perform Subclinical AF Detection?

*Answer: The implementation of AF screening in the VA diagnostic approach remains an unmet need.*

For many patients, measurement of VA biomarkers with automated devices might represent a unique opportunity to effectively diagnose AF. It could be supposed that individuals currently prescribed VA assessment for clinical purposes are individuals with a high burden of CV risk factor and therefore at high risk of developing AF. In these patients, targeted AF screening and early AF detection could potentially prevent the risk of ischemic stroke and AF-related complications [4]. Despite the potential benefits and the relatively simple technological advances needed for implementation, there are no data regarding the performance of devices measuring VA in AF detection.

Photopletysmogram (PPG) signals are proposed as promising tools to assess VA. Indeed, the time taken for the PPG pulse wave to travel the arterial tree is a function of AS. Moreover, pulse wave shapes could reflect changes in VA [60]. Noteworthily, the detection of rhythm irregularities through the analysis of peripheral PPG signals is a promising application for AF detection in patients at risk [61]. However, at present, there is no device that combines these two technologies into a single apparatus. 

Devices using oscillometric techniques may also contribute to AF screening. Blood pressure monitors which detect AF from oscillometry-based algorithms have been in the market for a few years, with very high sensitivity and specificity rates, ranging from 90 to 100% [62]. The algorithm for diagnosis, based on pulse irregularity, could be implemented in devices measuring central blood pressure or pulse wave velocity from oscillometric cuffs.

### 3.5. Question 5—Does Atrial-Fibrillation-Related Rhythm Irregularity Have a Negative Impact on the Measurement of Vascular Age?

*Answer: From these limited available data (two studies), measurements of biomarkers of arterial structure during AF appear reliable. Results about reliability of FMD measurement during AF are, to date, inconclusive.*

In a clinical study, cfPWV and cPP assessed by applanation tonometry were estimated in 34 patients with AF before and after successful electrical cardioversion [20]. After adjustment for post-procedural changes in mean arterial pressure (MAP) and heart rate, the intra-class correlation coefficient for both cfPWV and cPP was 0.89 (95%CI 0.79–0.95 for cfPWV, 0.72–0.95 for cPP), consistent with good reliability [63]. By contrast, measures of wave reflection such as central augmentation index (AIx) showed only moderate reliability (ICC = 0.59; 95%CI 0.17–0.80).

The reliability of FMD measurement was assessed in 32 patients with AF by comparing measures obtained before and 24 h after successful electrical cardioversion [21]. In this study, reliability was not formally tested using ICC but using a 2-sided *t*-test for independent samples, with further calculation of 95% confidence intervals from a Bland–Altman plot. As compared with FMD measurement taken during AF rhythm, FMD 24 h after restoration of sinus rhythm was, on average, significantly higher and showed high heterogeneity (FMD during AF rhythm 8.4 ± 3.8%, FMD 24 h after sinus rhythm restoration 10.7 ± 3.9%, *p* < 0.001, 95%CI for mean difference 1.15–3.65). The lack of appropriate statistics to assess reliability and the large time difference do not allow making definite conclusions about reliability of FMD during AF rhythm.

## 4. Discussion

In this paper, we aimed to review and summarize state-of-the-art data from the literature exploring the potential link between mechanical and functional biomarkers of VA and the presence and severity of AF. We aimed to find answers to five clinically and methodologically relevant questions and identify open and unanswered issues, including the cross-sectional association between VA and AF, the predictive role of VA for AF incidence, the role of VA as a risk enhancer for CV events in AF patients, the accuracy of VA measurement in AF rhythm and the performance of devices measuring VA in detecting AF rhythm.

This review was conceived as part of the work plan of the VascAgeNet COST action (COST Action CA18216) which is to refine, harmonize and promote the VA concept, to bring innovations in CV research from bench to bedside and to establish assessment of VA in clinical practice [19]. 

Our results showed that VA has a cross-sectional relationship with AF and is also independently associated with increased risk of incident AF. This was found for several biomarkers of arterial structure, such as PWV as a proxy measure of AS, wave reflection, arterial distensibility and VA-related measures of central hemodynamics such as central PP. A visual summary of the degree of the association between each measure of VA according to methodology questions is provided in Figure 2. Although less pronounced, there is substantial evidence of an independent link between VA biomarkers related to arterial function, such as brachial FMD, and AF.

The relationship between VA and AF has a profound rationale and is supported by shared common etiological mechanisms, such as elevated BP values and several other CV risk factors. Since BP is a surrogate marker of cardiac and arterial load, it is important to identify which temporal relationship exists between increased AS, elevated BP and AF and whether or at which level this process could be reverted by therapeutic approaches. There is robust evidence to support the hypothesis that increased arterial stiffness could precede the pathogenesis of elevated BP [64]. Therefore, measurement of arterial stiffness and VA in clinical practice could be the first ideal screening step to tackle the AF burden by identifying and targeting interventions in individuals with elevated AS at risk of developing hypertension and subsequent CV events, including AF. A further factor possibly influencing the link between FA and arterial stiffness is that both contribute to blood pressure variability, which is a potential independent risk factor for cardiovascular complications. Both the fluctuations of blood pressure induced by FA and the aging of vessels may cause an increased blood pressure variability in the short term [65]. In turn, an increased blood pressure variability is associated with an increased incidence of FA [66] and with cardiovascular outcomes among patients with FA [67], making this association a topic of major interest for future research. 

The promotion of a healthier lifestyle early in life through increasing physical activity, healthy diet, smoking cessation, weight control, lowering stress and normalization of sleep patterns was found to be associated with lower levels of arterial stiffness [61]. There is also strong evidence that AF has an independent association with ED, measured by FMD of the brachial artery. However, this association seems to be largely explained by concomitant CV risk factors, such as hypertension, dyslipidemia and diabetes, which are known to negatively affect the endothelial function, and it is currently unknown whether ED could be reverted by therapeutic approaches. However, to our knowledge, no study has investigated the role of more stringent therapeutic goals aiming at a better VA control on AF-related outcomes.

Even though increased arterial stiffness has been described as a predictor of AF, available evidence regarding its prognostic value for CV events in patients with AF is far from conclusive. This is due to the lack of relevant data, often originating from underpowered studies with high heterogeneity in terms of arterial stiffness markers, as well as to the methods for assessing AS. To this end, larger, prospective, community-based cohorts with longer follow-up periods are needed. The prognostic value of ED for future CV events is understudied in AF patients and further studies are needed specifically targeting this population.

At the methodological level, there are still few data regarding the performance of devices measuring arterial stiffness in detecting AF. Given that a considerable proportion of patients with AF are undiagnosed, developing technology would help increase the screening and the detection rates of this condition. The potential outcome of combining screening approaches for the evaluation of VA and AF detection into one single device needs therefore to be tested in future dedicated studies. The measurement of VA biomarkers during irregular cardiac rhythm, as observed in AF with irregular response rate, represents a practical challenge that is not fully overcome. As a consequence, AF patients are often excluded from clinical trials with VA assessment. We showed that, despite preliminary promising results provided by a few methodological studies, substantial research should focus on technological solutions addressing this issue.

## 5. Conclusions

In conclusion, given the close pathophysiological link between VA and AF, it is reasonable that measurements of arterial stiffness should be implemented in clinical practice in all individuals at risk of developing AF and its adverse consequences to better stratify their risk. The predictive role of both arterial stiffness and ED as CV risk factors in AF patients still needs to be proven in dedicated studies. Future studies and upcoming technologies will be helpful to address the gap of knowledge in this field.

## Figures and Tables

**Table 1:** Description of vascular aging biomarkers.

Vascular Aging Biomarker	Method of Measurement
Carotid–femoral pulse wave velocity (cfPWV)	Ratio of traveled distance between the carotid and femoral pulse site and transit time between common carotid and common femoral artery; based on tonometers, piezoelectronic sensors, cuffs or Doppler ultrasound, either simultaneously or sequentially, using ECG for gating.
Heart–femoral pulse wave velocity (hfPWV)	Ratio of traveled distance between the heart and femoral pulse sites and transit time starting from second heart sound; based on tonometers, ECG and microphones.
Brachial–ankle pulse wave velocity (baPWV)	Ratio between traveled distance and transit time calculated with occlusive cuffs placed at brachial artery and ankle; cardio-ankle vascular index is a variation using a phonocardiogram and occlusive cuffs.
Arterial stiffness index (ASI)	Marker of arterial stiffness calculated by dividing height by the timing of reflected waves from finger photoplethysmography
Cardio-ankle vascular index (CAVI)	Marker of arterial stiffness based on the stiffness parameter β, reflecting arterial properties from origin of the ascending aorta to the ankle.
Brachial pulse pressure (PP)	Measured using validated sphygmomanometers; brachial pulse pressure defined as systolic minus diastolic BP.
Central pulse pressure (cPP)	Central pulse pressure based on waveforms recorded at the radial, brachial or carotid artery, mainly using tonometers or cuffs; waveforms are calibrated with measured brachial BP leading to central systolic BP and pulse pressure.
Augmentation index (AIx)	The ratio between central augmented pressure and pulse pressure, as a surrogate indicator of wave reflections and left ventricular loading.
Pulse pressure amplification (PPA)	Central to peripheral pulse pressure amplification (peripheral PP/central PP) is due to both cardiac and arterial factors: ventricular ejection, arterial stiffness, amplitude and timing of wave reflection. VA reduces PPA values.
Brachial artery flow-mediated dilation (FMD)	Flow-mediated dilation induces the release of nitric oxide, resulting in vasodilation that can be measured by ultrasound imaging of the diameter of the brachial artery after an ischemia induced by arterial occlusion using a cuff, which is released after 5 min, leading to reactive hyperemia.
Aortic distensibility	Measure of aortic elasticity estimated by the relative change in diameter, area or volume divided by the pulse pressure generating this change; may be measured by echocardiography or by MRI.
Carotid artery distensibility	Measure of carotid artery elasticity estimated by the ratio between relative change in diameter or volume and the pulse pressure generating this change; usually measured by carotid ultrasound.

**Table 2:** Summary of results from clinical studies in adult humans organized according to methodology questions.

Year, Author, Country	Method (Biomarker)	Population	Study Design	Main Results	Questions				
					1	2	3	4	5
2018, Caluwé R et al., Belgium [20]	SphygmoCor (cfPWV, AIx, central pulse pressure)	34 patients with AF	Experimental study: before and after cardioversion for AF	Good agreement before and after cardioversion for cfPWV and cPP, moderate agreement for AIx.					
2007, Skalidis EI et al., Greece [21]	Brachial artery FMD and NMD (FMD, NMD)	46 patients with AF and 25 controls	Experimental study, before and after electrical cardioversion	FMD improved after successful cardioversion, while NMD was not significantly altered. High agreement in Bland–Altmann analysis.					
2016, Chen SC et al., Taiwan [22]	Omron VP-1000 (baPWV)	167 patients with AF	Longitudinal observational study	In patients with AF, a high baPWV was independently associated with increased CV events.					
2021, Shchetynska-Marinova T et al., Germany [23]	Echocardiography (aortic distensibility)	151 patients with AF and 54 controls	Longitudinal observational study	AF was associated with reduced aortic distensibility, left atrial size and pulse pressure. The incidence of AF recurrences increased with loss of aortic distensibility.					
2011, Chen LY et al., USA [24]	SphygmoCor (cfPWV)	118 patients with AF, 274 controls	Observational. case-control study	CfPWV was associated with NT-proBNP level in AF.					
2015, Perri L et al., Italy [25]	Brachial artery FMD (FMD)	514 non-valvular AF patients	Experimental prospective study	In patients with AF, low FMD (<4.6%) independently predicted CV events.					
2022, Zhang J et al., China [26]	Brachial artery FMD (FMD)	291 with paroxysmal AF	Longitudinal observational study	FMD was a predictor of CV events in patients with PAF.					
2016, Chen LY et al., USA [27]	IMT, carotid distensibility (Echodoppler), aortic PWV (Complior)	13,907 ARIC, 6640 MESA, 5220 Rotterdam Study	Longitudinal observational study	Higher IMT and greater arterial stiffness were associated with higher AF incidence, with modest improvement in AF risk prediction.					
2021, Almuwaqqat Z et al., USA [28]	Omron VP-1000 (cfPWV)	3882 elderly participants of ARIC	Longitudinal observational study	Low (first quartile) and high (third and fourth quartiles) cfPWV were associated with higher AF risk.					
2016, Shaikh AY et al., USA [29]	Arterial tonometry (cfPWV, CPP, AIx). Doppler ultrasound (FMD)	5797 Framingham tonometry sample; 3921 ED sample	Retrospective observational study	Higher AIx and central pressure, lower FMD were associated with increased risk of incident AF.					
2022, Nagayama D et al., Japan [30]	VaSera cardio-ankle vascular index (CAVI)	47,687 cross-sectional study; 5418 cohort study	Cross-sectional and longitudinal study	CAVI was independently associated with AF. CAVI ≥8.0 was an independent predictor for AF incidence.					
2014, Roetker NS et al., USA [31]	Brachial oscillometry, MRI (pulse pressure, aortic distensibility)	6630 participants from the MESA	Longitudinal observational study	Higher levels of systolic and pulse pressure were associated with increased risk of AF. Aortic distensibility was not consistently associated with the risk of AF.					
2012, Valbusa F et al., Italy [32]	Brachial oscillometry (pulse pressure)	350 patients with type 2 diabetes mellitus	Longitudinal observational study	Increased pulse pressure independently predicted incident AF in 10-year follow-up.					
2012, Larstorp AC et al., Norway [33]	Oscillometry (pulse pressure)	8810 hypertensive patients	Longitudinal observational study	Pulse pressure was the strongest single BP predictor of new-onset AF.					
2007, Mitchell G et al., USA [34]	Brachial oscillometry (pulse pressure)	5331 Framingham Heart Study participants initially free from AF	Longitudinal observational study	Pulse pressure was associated with increased risk for AF (adjusted hazard ratio, 1.26 per 20 mm Hg increment; *p* = 0.001).					
2021, Matsumoto K et al., USA [35]	ABPM, SphygmoCor (central BP, ambulatory BP)	769 participants in sinus rhythm	Longitudinal observational study	ABPM was a better independent predictor of incident AF than central BP.					
2022, Shchetynska-Marinova T et al., Germany [36]	Echocardiography (aortic distensibility)	151 patients with AF who underwent pulmonary vein isolation	Longitudinal observational study	Reduced aortic distensibility and increased atrial size were associated with AF recurrence.					
2013, Lau DH et al., Australia [37]	SphygmoCor (Central BP, AIx)	68 patients with lone AF undergoing successful catheter ablation	Longitudinal observational study	Central pulse pressure ≥ 45 mmHg and augmentation pressure ≥ 12 mmHg were both associated with lower survival free from AF.					
2016, Fumagalli S et al., Italy [38]	VaSera Cardio-ankle vascular index (CAVI)	31 patients with AF	Longitudinal observational study	After cardioversion, AF persistence at follow-up was associated with higher CAVI.					
2015, Kizilirmak F et al., Turkey [39]	Mobil-O-Graph (central pulse pressure, PWV, AIx)	103 patients with PAF, 103 controls	Longitudinal observational study	Increased arterial stiffness markers were associated with AF occurrence but not predicted recurrence after catheter ablation.					
2019, Zekavat SM et al., USA [40]	Finger photoplethysmography (ASI)	225,636 UK Biobank participants	Genome-wide association study. Mendelian randomization	Genetic predisposition to higher ASI was significantly associated with increased risk of incident and prevalent AF					
2010, Drager LF et al., Portugal [41]	SphygmoCor, echocardiography (cf-PWV, left atrial diameter)	73 middle-aged patients	Observational study	Left atrial diameter is associated with pulse wave velocity independently of common determinants.					
2008, Lantelme P et al., France [42]	Complior, ABPM, echocardiography (cfPWV, 24 h PP, left atrial diameter)	310 hypertensive patients	Observational study	Left atrial diameter is associated with cfPWV and 24 h PP independently from classical determinants (e.g., age, BMI, LV dimensions and geometry).					
2021, Garg PK et al., USA [43]	Brachial artery FMD (FMD)	2027 elderly patients	Longitudinal observational study	The risk of incident AF was not dependent on baseline FMD when analysis was adjusted for confounders.					
2021, Pauklin P et al., Estonia [44]	Oscillometry, SphygmoCor (cfPWV, central BP, pressure amplification)	76 patients with AF	Observational study	Patients with AF had significantly higher cSBP, cPP, PWV compared to healthy controls. Positive correlation of left atrial diameter and volume with PWV.					
2017, Cui R et al., Japan [45]	Omron HEM9000AI (AIx)	4264 participants	Longitudinal observational study	AIx values, but not brachial or central pulse pressures, were positively and independently associated with the prevalence of AF.					
2009, Doi M et al., Japan [46]	Omron HEM9000AI (radial Aix)	122 patients with PAF (in sinus rhythm), 122 controls	Observational Case–control study	AIx was significantly higher in patients with PAF than in subjects without PAF.					
2005, Shi D et al., China [47]	Omron VP-1000 (brachial–ankle PWV)	132 patients with hypertension and AF (78 paroxysmal, 84 persistent) and 136 with only hypertension	Observational study	Patients with AF and hypertension presented higher baPWV values than hypertension alone. Persistent AF was associated with higher baPWV than PAF.					
2008, Lee SH et al., Republic of Korea [48]	VP-2000 (heart–femoral PWV)	35 subjects with sinus rhythm, 33 subjects with AF	Observational case–control study	Patients with AF had higher hfPWV than those in sinus rhythm. AF was an independent predictor of increased hfPWV together with age and systolic BP.					
2014, Miyoshi T et al., Japan [49]	VaSera cardio-ankle vascular index (CAVI)	91 patients with PAF compared with 90 matched controls	Case–control study	CAVI was significantly higher in patients with PAF than in controls.					
2014, Fumagalli S et al., Italy [50]	VaSera cardio-ankle vascular index (CAVI)	33 patients with AF	Observational study	CAVI obtained immediately after cardioversion was associated with short AF duration and left atrial diameter.					
2021, Chung GE et al., Korea [51]	VaSera cardio-ankle vascular index (CAVI)	8048 subjects	Longitudinal observational study	High CAVI was associated with AF in those with intermediate or high CV risk.					
2020, Heshmat-Ghahdarijani K et al., Iran [52]	Brachial artery FMD (FMD)	43 patients with AF and 51 controls	Case–control study	FMD of patients with AF was significantly lower than controls.					
2004, Guazzi M et al., Italy [53]	Brachial artery FMD	35 patients with lone AF undergoing external cardioversion	Longitudinal observational study	Brachial FMD improved after cardioversion and returned to basal values in subjects with AF recurrency.					
2019, Börschel CS et al., Germany [54]	Brachial artery FMD and peripheral arterial tonometry (PAT ratio)	15,010 subjects (466 AF)	Observational study	FMD and PAT were compromised in individuals with AF, but associations were mediated by age and classical risk factors.					
2021, Khan AA et al., United Kingdom [55]	Brachial artery FMD	30 patients with permanent AF vs. 31 patients with PAF	Case–control study	Duration and frequency of AF lead to worsening endothelial function.					
2013, Polovina M et al., Serbia [56]	Brachial artery FMD and NMD	38 patients with persistent AF and 28 controls	Observational case–control study	FMD of AF patients was significantly lower than FMD of healthy controls. No differences in median NMD values.					

Questions: 1. Correlation of AF and VA. 2. Prediction of AF. 3. Prognostic value of VA in AF. 4. Detection of AF with devices. 5. Accuracy of measurement of VA in AF. Colors legend: Dark green: Significant association in a prospective study. Light green: Significant association. Yellow: Neutral or absent association. Abbreviations: ABPM: ambulatory blood pressure monitoring. ARIC: Atherosclerosis Risk in Communities, MESA: Multi-Ethnic Study of Atherosclerosis. Complete list of abbreviations at the end. Instruments: SphygmoCor (ATCOR, Sidney, Australia), Omron VP-1000, VP-2000 and HEM9000AI (Omron, Kyoto, Japan), VaSera (Fukuda Denshi, Tokyo, Japan), Mobil-O-Graph (IEM GmbH, Aachen, Germany).

## Supplementary Materials

The following supporting information can be downloaded at: https://www.mdpi.com/article/10.3390/jcm13051207/s1, Table S1: Narrative review checklist.
