# Supplementary material for: Atrial Fibrillation and Early Vascular Aging: Clinical Implications, Methodology Issues and Open Questions—A Review from the VascAgeNet COST Action
Source: J Clin Med. 2024 Feb 20;13(5):1207. doi: 10.3390/jcm13051207 (PMC10931681; doi:10.3390/jcm13051207)
Supplement: Supplementary file 1 [file jcm-13-01207-s001.zip › jcm-2839175-supplementary.pdf]

Table S1. Narrative review checklist (From https://legacyfileshare.elsevier.com/promis_misc/ANDJ%20Narrative%20Review%20Checklist.pdf).

Section/Topic	#	Checklist Item	Reported on Page
		**TITLE**	
Title	1	Identify the report as a narrative review	1
		**ABSTRACT**	
Unstructured summary	**2**	Provide an unstructured summary, including, as applicable: background. Objective, brief summary of narrative review and implications for future research, and clinical practice or policy development.	**1**
		**INTRODUCTION**	
Rationale/background	**3**	Describe the rationale for the review in the context of what is already known.	**2**
Objectives	**4**	Specify the key question(s) for the review topic.	**3**
		**METHODS**	
Research selection	**5**	Specify the process for identifying the literature search (e.g., years considered, language, publication status, study design, and databases of coverage).	**5**
		**DISCUSSION/SUMMARY**	
Narrative	**6**	Discuss: (1) research reviewed, including fundamental or key findings, (2) limitations and/or quality of research reviewed, and (3) need for future research.	**6-17**
Summary	**7**	Provide and overall interpretation of the narrative review in the context of clinical practice for health professionals, policy development and implementation, or future research.	**18-19**
